# Supplementary material for: Lymphoid B cells upregulate HIV-1 ex vivo and are linked to its expression in vivo
Source: PLoS Pathog. 2025 Dec 1;21(12):e1013661. doi: 10.1371/journal.ppat.1013661 (PMC12680345; doi:10.1371/journal.ppat.1013661)
Supplement: S1 Fig — (A) Dose response curves for GCB-induced HIV expression. 2x10^5^ TFH were spinoculated with X4-HIV and cultured with autologous uninfected CellTrace Blue labeled GCB at the ratios indicated. Percent GFP+ TFH and GFP MFI of GFP+ TFH were determined by flow cytometry. These data were used to determine fold differences as reported in Fig 1E. (B) Cell counts of uninfected, CellTrace labeled TFH and GCB after 3 days in culture with HIV spinoculated TFH as reported in Fig 1H. (PDF) [file ppat.1013661.s001.pdf]

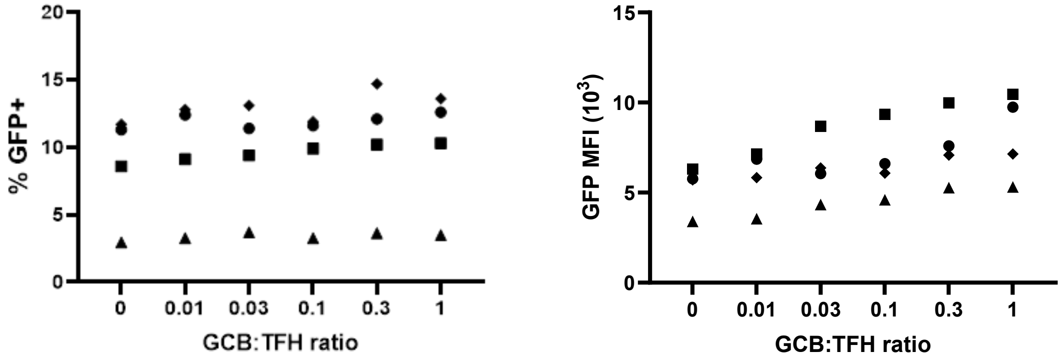

Figure S1. Dose response curves for GCB-induced HIV expression. $2x10^5$ TFH were spinoculated with X4-HIV and cultured with uninfected CellTrace Blue labeled GCB at the ratios indicated. Percent GFP+TFH and GFP MFI of GFP+TFH were determined by flow cytometry. These data were used to determine fold differences as reported in Fig. 1E.
