# Supplementary material for: Lymphoid B cells upregulate HIV-1 ex vivo and are linked to its expression in vivo
Source: PLoS Pathog. 2025 Dec 1;21(12):e1013661. doi: 10.1371/journal.ppat.1013661 (PMC12680345; doi:10.1371/journal.ppat.1013661)
Supplement: S2 Fig — (A) Upregulation of GFP expression in TFH (CD3+CD8-CD19-CXCR5hiPD-1hi) by GCB (CD19+CD3-CD38midIgD-) for experiments reported in Figs 2B and S2D. (B-D) Volcano plots depicting differentially expressed genes from (B) R5-HIV GFP reporter virus (R5-HIV) spinoculated TFH cultured with uninfected, dye labeled TFH (R5-HIV TFH + TFH) compared to mock spinoculated TFH cultured with dye-labeled TFH (Mock TFH + TFH), (C) R5-HIV spinoculated TFH cultured with dye-labeled GCB (R5-HIV TFH + GCB) compared to mock spinoculated TFH cultured with dye-labeled GCB (Mock TFH + GCB), and (D) X4-HIV spinoculated TFH cultured with GCB (X4-HIV TFH + GCB) compared to X4-HIV spinoculated TFH (X4-HIV TFH). Vertical lines represent 1.5 fold change and horizontal lines represent an adjusted p value of 0.05, as determined using the Benjamini Hochberg method. (E) Heatmap of the 126 genes altered significantly in at least one of the three cultures reported in Figs 2A, 2B and S2D. (F) The number of TFH genes significantly altered by GCB that were shared or distinct in X4-HIV (grey), R5-HIV (green), or Mock (pink) cultures as reported in (D), Fig 2B and 2A, respectively. (G) TFH were sorted from CD4 enriched tonsil cells in which CD3 antibody was either included (CD3+CD8-CD19-CXCR5hiPD-1hi) or omitted (CD8-CD19-CXCR5hiPD-1hi) in autologous preparations. TFH were spinoculated with X4-HIV and cultured with uninfected labeled TFH or GCB for 3 days in R-15 and 5μM saquinavir. GCB-mediated fold differences in percentages of GFP+ TFH (top) and GFP MFI of GFP+ TFH (bottom) were determined (n = 6). (H-L) TFH isolated from tonsils were spinoculated with X4-HIV and cultured with uninfected, CellTrace Blue labeled TFH or GCB at a ratio of 1:1. Cells were cultured in the presence or absence of (H) soluble ICOS, sCD40L, or blocking antibody to CD40 (n = 6), (I) with or without blocking antibody to ICAM-1 (n = 4), (J) in the presence or absence of neutralizing antibody to IL-2, GITRL, or 4–1BB-Fc (n = 3), (K) DMS [file ppat.1013661.s002.pdf]

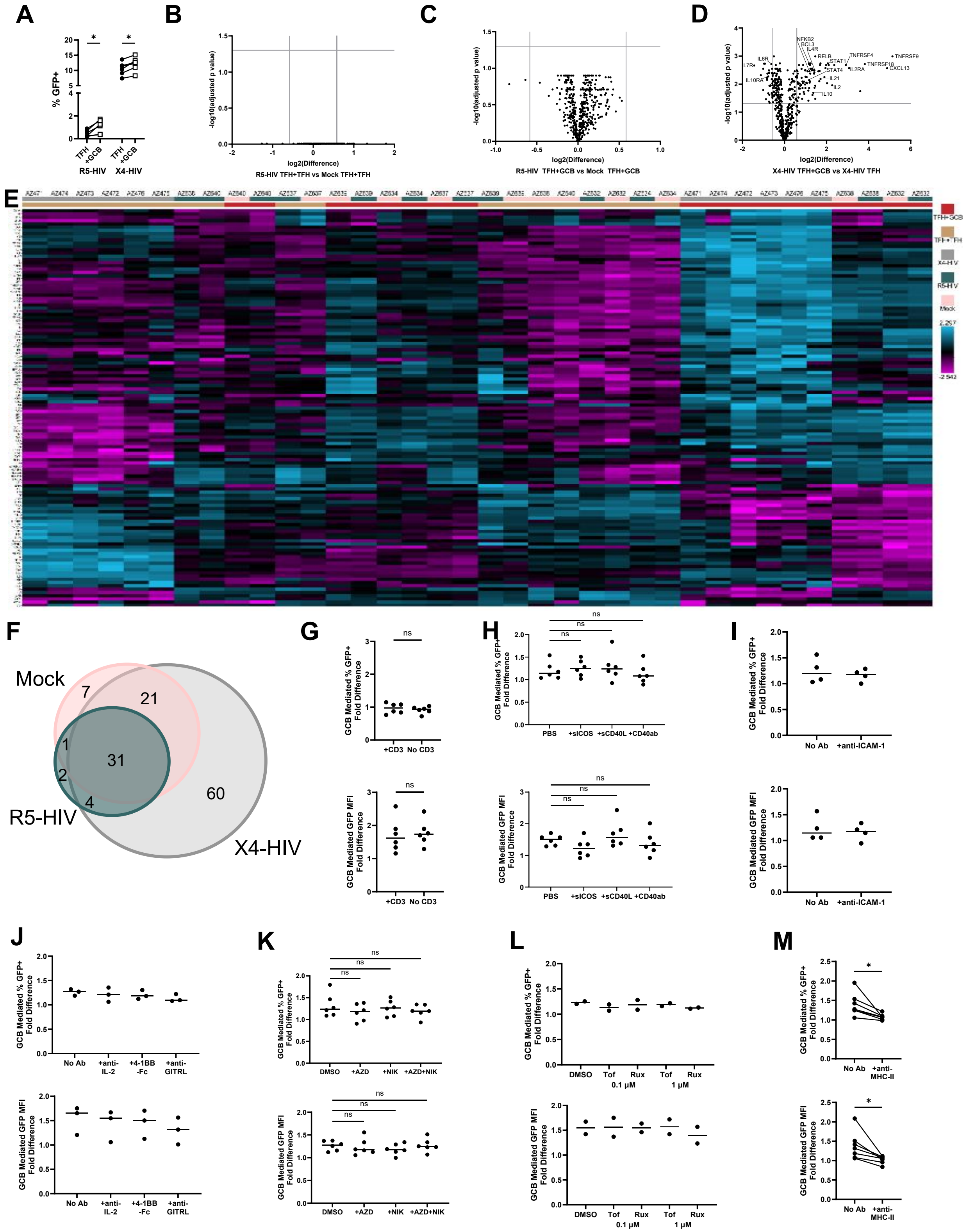

Figure S2. Gene expression analyses of cultures of TFH with either TFH or GCB and examination of several mechanisms by which GCB could potentially upregulate HIV

expression in TFH. (A) Upregulation of GFP expression in TFH (CD3+CD8-CD19-CXCR5hiPD-1hi) by GCB (CD19+CD3-CD38midIgD-) for experiments reported in Fig. 2B and Fig. S2D. (B-D) Volcano plots depicting differentially expressed genes from (B) R5-HIV GFP reporter virus (R5-HIV) spinoculated TFH cultured with uninfected, dye labeled TFH (R5-HIV TFH+TFH) compared to mock spinoculated TFH cultured with dye-labeled TFH (Mock TFH+TFH), (C) R5-HIV spinoculated TFH cultured with dye-labeled GCB (R5-HIV TFH+GCB) compared to mock spinoculated TFH cultured with dye-labeled GCB (Mock TFH+GCB), and (D) X4-HIV spinoculated TFH cultured with GCB (X4-HIV TFH+GCB) compared to X4-HIV spinoculated TFH (X4-HIV TFH). Vertical lines represent 1.5 fold change and horizontal lines represent an adjusted p value of 0.05, as determined using the Benjamini Hochberg method. (E) Heatmap of the 126 genes altered significantly in at least one of the three cultures reported in Fig. 2A-B, Fig. S2D. (F) The number of TFH genes significantly altered by GCB that were shared or distinct in X4-HIV (grey), R5-HIV (green), or Mock (pink) cultures as reported in (D), Fig. 2B, and Fig. 2A, respectively. (G) TFH were sorted from CD4 enriched tonsil cells in which CD3 antibody was either included (CD3+CD8-CD19-CXCR5hiPD-1hi) or omitted (CD8-CD19-CXCR5hiPD-1hi) in autologous preparations. TFH were spinoculated with X4-HIV and cultured with uninfected labeled TFH or GCB for 3 days in R-15 and 5μM saquinavir. GCB-mediated fold differences in percentages of GFP+TFH (top) and GFP MFI of GFP+TFH (bottom) were determined (n=6). (H-L) TFH isolated from tonsils were spinoculated with X4-HIV and cultured with uninfected, CellTrace Blue labeled TFH or GCB at a ratio of 1:1. Cells were cultured in the presence or absence of (H) soluble ICOS, sCD40L, or blocking antibody to CD40 (n=6), (I) with or without blocking antibody to ICAM-1 (n=4), (J) in the presence or absence of neutralizing antibody to IL-2, GITRL, or 4-1BB-Fc (n=3), (K) DMSO, 0.1μM of the noncanonical NFκB activator AZD5582, 5μM of the non-canonical NFκB inhibitor NIK-SMI1, or both AZD5582 and NIK-SMI1 (n=6), or (L) DMSO, and either 0.1μM or 1μM of JAK inhibitors tofacitinib (Tof) or ruxolitinib (Rux) (n=2). After 3 days, percent CellTrace Blue-GFP+TFH and GFP MFI of CellTrace Blue-GFP+TFH were determined by flow cytometry and reported as GCB-mediated fold differences. (M) GCB mediated fold differences of experiments as reported in Fig. 2D. Horizontal bars indicate medians (G-L). Statistical analyses of (A,G,H,K,M) were determined using Wilcoxon matched paired tests as determined by Graphpad Prism v10 and significance indicated: ns, not significant; *$p<0.05$.
