# Supplementary material for: Lymphoid B cells upregulate HIV-1 ex vivo and are linked to its expression in vivo
Source: PLoS Pathog. 2025 Dec 1;21(12):e1013661. doi: 10.1371/journal.ppat.1013661 (PMC12680345; doi:10.1371/journal.ppat.1013661)
Supplement: S3 Fig — (PDF) [file ppat.1013661.s003.pdf]

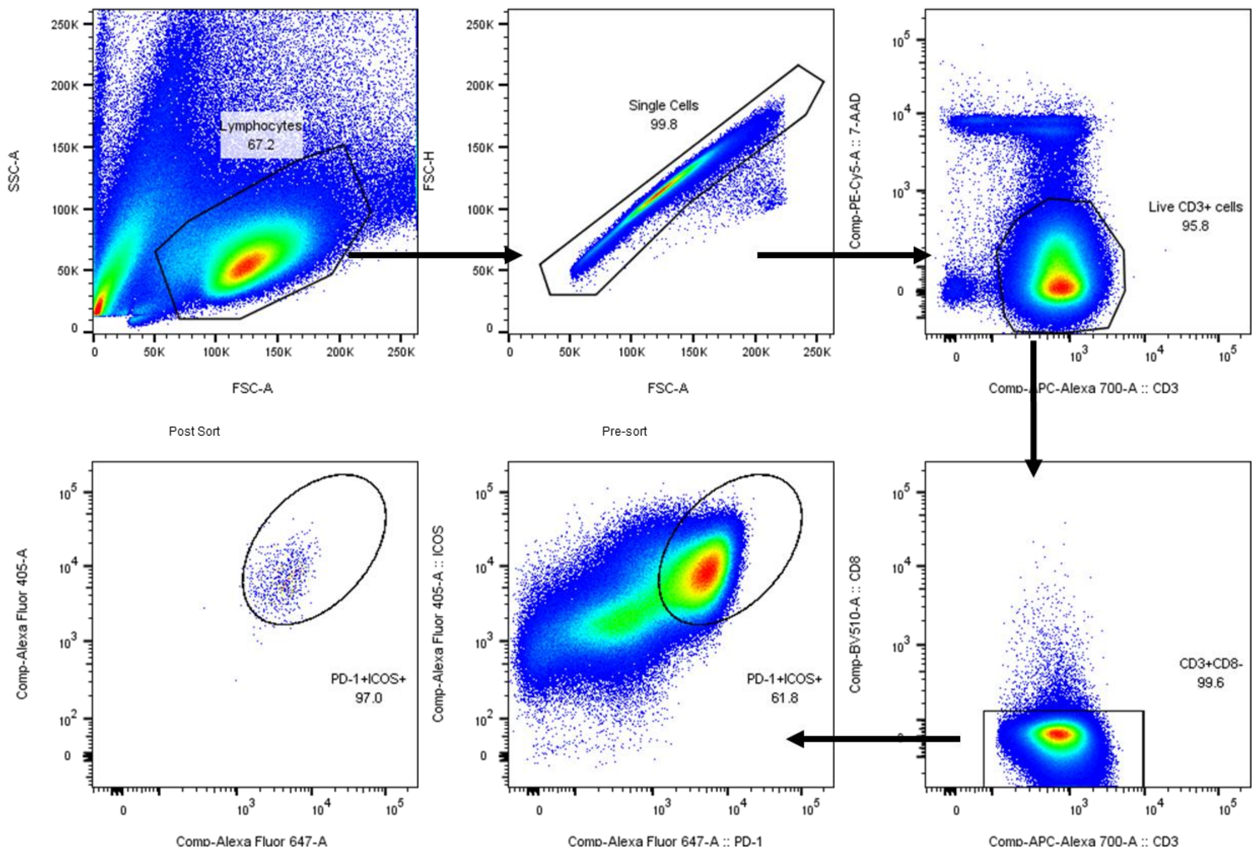


**Figure S3. Representative gating strategy for isolation of TFH by FACS in which ICOS antibody was included and CXCR5 antibody omitted as used in Fig. 3D**.
