# Supplementary material for: Lymphoid B cells upregulate HIV-1 ex vivo and are linked to its expression in vivo
Source: PLoS Pathog. 2025 Dec 1;21(12):e1013661. doi: 10.1371/journal.ppat.1013661 (PMC12680345; doi:10.1371/journal.ppat.1013661)
Supplement: S4 Fig — (A) 4x10^6^ nonTFH spinoculated with R5-HIV GFP reporter virus were cultured with 4x10^6^ uninfected, violet proliferation dye labeled nonTFH (R5-HIV nonTFH + nonTFH) or nonGCB (R5-HIV nonTFH + nonGCB) for 3 days in R-15 with 5μM saquinavir. Live, violet proliferation dye- nonTFH were isolated by FACS and total RNA was purified. Gene expression analysis was performed using Nanostring’s host response panel. Volcano plot depicts differentially expressed genes in R5-HIV nonTFH + nonGCB when compared to R5-HIV nonTFH + nonTFH. Vertical lines represent 1.5 fold change and horizontal lines represent an adjusted p value of 0.05, as determined using the Benjamini Hochberg method. (n = 6) (B) Directed global significance scores were determined for the 30 pathways in which ≥15 genes from the host response panel were assigned using nSolver 4.0 advanced analysis. (C) The number of genes significantly altered by B cells that were shared or distinct in R5-HIV nonTFH (blue), Mock TFH (pink), R5-HIV TFH (green), and X4-HIV TFH (grey) cultures as reported in (A), Figs 2A, 2B, and S2D, respectively. (PDF) [file ppat.1013661.s004.pdf]

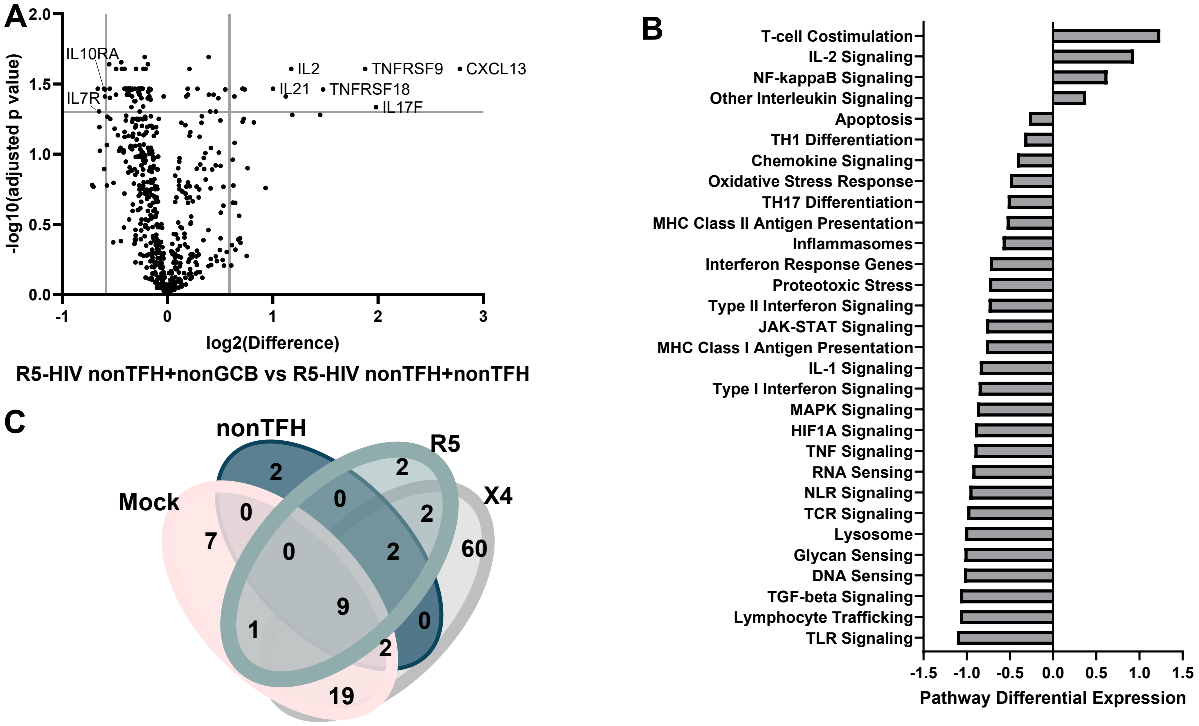

Figure S4. SLT B cells upregulate HIV expression in TFH and nonTFH and alter gene expression in nonTFH. (A) $4x10^6$ nonTFH spinoculated with R5-HIV GFP reporter virus were cultured with $4x10^6$ uninfected, violet proliferation dye labeled nonTFH (R5-HIV nonTFH+nonTFH) or nonGCB (R5-HIV nonTFH+nonGCB) for 3 days in R-15 with 5µM saquinavir. Live, violet proliferation dye- nonTFH were isolated by FACS and total RNA was purified. Gene expression analysis was performed using Nanostring's host response panel. Volcano plot depicts differentially expressed genes in R5-HIV nonTFH+nonGCB when compared to R5-HIV nonTFH+nonTFH. Vertical lines represent 1.5 fold change and horizontal lines represent an adjusted p value of 0.05, as determined using the Benjamini Hochberg method. (n=6) (B) Directed global significance scores were determined for the 30 pathways in which ≥15 genes from the host response panel were assigned using nSolver 4.0 advanced analysis. (C) The

number of genes significantly altered by B cells that were shared or distinct in R5-HIV nonTFH (blue), Mock TFH (pink), R5-HIV TFH (green), and X4-HIV TFH (grey) cultures as reported in (A), Fig. 2A-B, and Fig. S2D, respectively.
