# Supplementary material for: Lymphoid B cells upregulate HIV-1 ex vivo and are linked to its expression in vivo
Source: PLoS Pathog. 2025 Dec 1;21(12):e1013661. doi: 10.1371/journal.ppat.1013661 (PMC12680345; doi:10.1371/journal.ppat.1013661)
Supplement: S5 Fig — (A) Representative images of inguinal lymph node sections illustrating vRNA+ TFH defined as DAPI+vRNA+BCL6+PD-1+ inside (top) and outside (bottom) B cell follicles, as determined by CD20 staining (not shown). All in situ targets were imaged using z-stacks and shown as compressed images. (B) Percentages of vRNA+DAPI+ cells expressing BCL6, PD-1, and both in PLWH. (C) Representative single color images of the same TFH stained by immunostaining for CD3 (top left), PD-1 (bottom left), and in situ hybridization for CD4 (top right), and BCL6 (bottom right). DAPI (blue) was used to identify nuclei. Arrows indicate the same TFH cell. (D) Frequencies of CD3+CD4+ T cells were determined in follicles (F) and extrafollicular (EF) regions in PLWH. Horizontal bars indicate medians (D). Mann-Whitney tests were performed for unpaired comparisons between PLWH on ART (+ART) and PLWH not on ART (No ART), and Wilcoxon tests were used for paired comparisons as determined by Graphpad Prism v10 and significance indicated: ns, not significant; *p < 0.05. (PDF) [file ppat.1013661.s005.pdf]

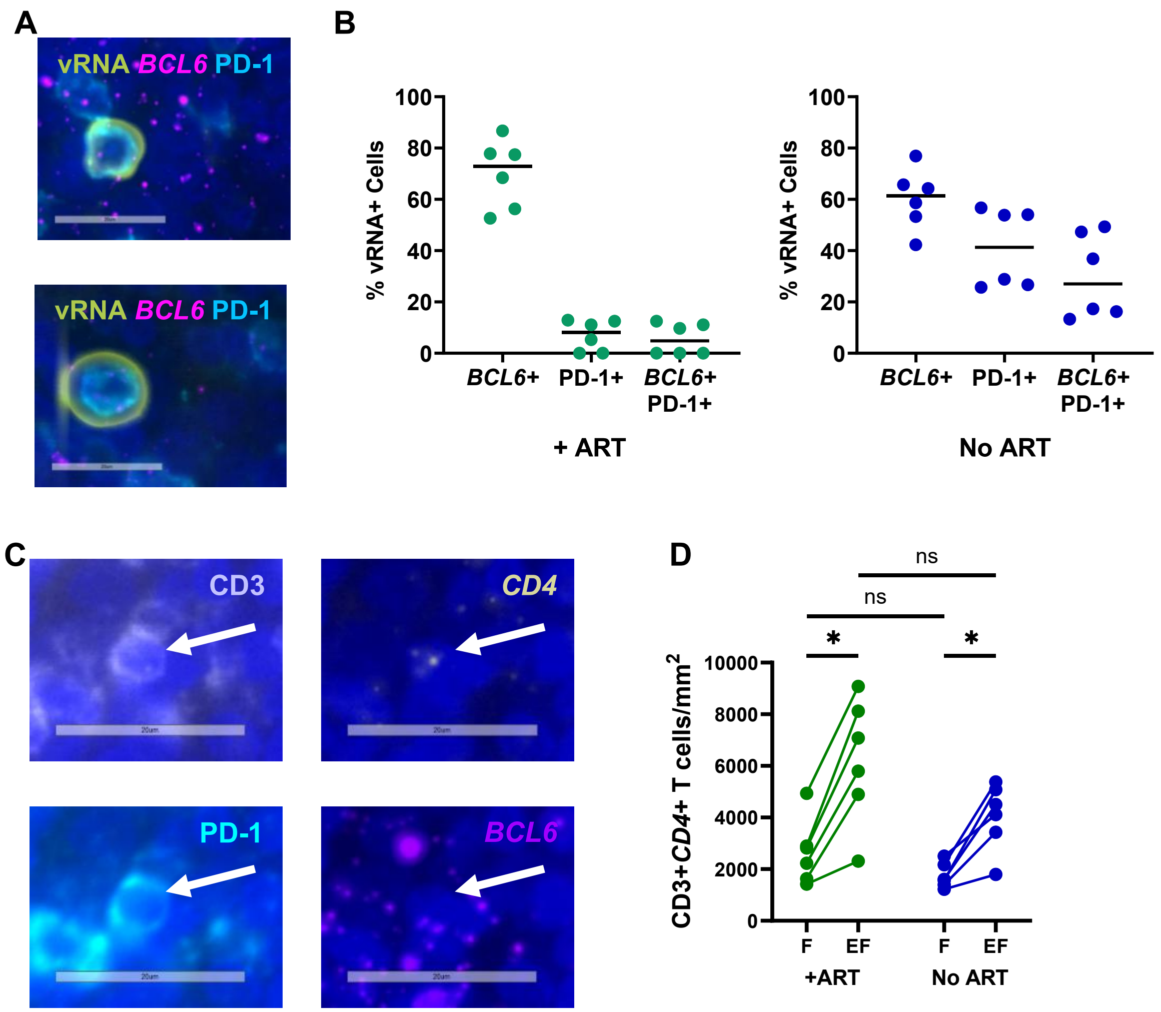

Figure S5. Representative staining for vRNA+TFH and TFH, frequencies of CD3+CD4+ cells within and outside of follicles, and frequencies of Bcl6+, PD-1+, and Bcl6+PD-1+ cells in lymph nodes from PLWH. (A) Representative images of inguinal lymph node sections illustrating vRNA+TFH defined as DAPI+vRNA+BCL6+PD-1+ inside (top) and outside (bottom) B cell follicles, as determined by CD20 staining (not shown). (B) Percentages of vRNA+DAPI+ cells expressing BCL6, PD-1, and both in PLWH. (C) Representative single color images of the same TFH stained by immunostaining for CD3 (top left), PD-1 (bottom left), and in situ hybridization for CD4 (top right), and BCL6 (bottom right). DAPI (blue) was used to identify nuclei. Arrows indicate the same TFH cell. (D) Frequencies of CD3+CD4+T cells were

determined in follicles (F) and extrafollicular (EF) regions in PLWH. Horizontal bars indicate medians (D). Mann-Whitney tests were performed for unpaired comparisons between PLWH on ART (+ART) and PLWH not on ART (No ART), and Wilcoxon tests were used for paired comparisons as determined by Graphpad Prism v10 and significance indicated: ns, not significant; *$p<0.05$.
