# Supplementary material for: Lymphoid B cells upregulate HIV-1 ex vivo and are linked to its expression in vivo
Source: PLoS Pathog. 2025 Dec 1;21(12):e1013661. doi: 10.1371/journal.ppat.1013661 (PMC12680345; doi:10.1371/journal.ppat.1013661)
Supplement: S1 Table — (PDF) [file ppat.1013661.s006.pdf]

Table S1. Numbers of vRNA+ cells evaluated from each donor

Subject ID	Number of vRNA+ cells analyzed	Number of vRNA+ cells analyzed for TFH phenotype
**PLWH: No ART**		
LN23	140	87
LN25	85	67
LN78	75	52
LN106	182	105
LN132	152	90
LN136	154	91
**PLWH: +ART**		
LN-01	21	9
LN-04	41	31
LN-05	25	15
LN-07	28	16
LN-08	33	19
LN-09	25	19
