# Supplementary material for: Lymphoid B cells upregulate HIV-1 ex vivo and are linked to its expression in vivo
Source: PLoS Pathog. 2025 Dec 1;21(12):e1013661. doi: 10.1371/journal.ppat.1013661 (PMC12680345; doi:10.1371/journal.ppat.1013661)
Supplement: Attachment — Submitted filename: The manuscript.docx [file ppat.1013661.s008.docx]

The manuscript “Lymphoid B cells upregulate HIV-1 ex vivo and 1 are linked to its expression in vivo” by Ollerton et all examines the impact of viral RNA expression in HIV-infected TFH by B cells. The authors use an in vitro model of HIV infection using cells derived from adolescent tonsils and ex vivo analysis of lymph nodes from adult PWH on or off ART. The authors characterize HIV expression in the presence or absence of GCB and use transcriptomics, pathway blockade and LRAs to probe the mechanisms. The authors conclude GCB increase the expression of HIV in TFH in a contact and MCH II dependent manner. The manuscript is well written, and the figures are clear.

Major points:

1. In Figure 2 the authors perform transcriptomics analysis on TFH cells in vitro infected with X4 or R5 tropic HIV in the presence or absence of GCB. Although the authors show the data in Figure 2 and report the gene expression measurements in xcel files, the authors seem to be leaving it up to the reader to review and interpret their findings. There is really no discussion of the findings in the manuscript other than to note a few genes in lines 128-131. The authors should include a more detailed discussion on the gene signatures they are showing in Figure 2 and more discussion on the significance of these findings in their conclusions. Do the authors observe any correlations between the magnitude of the change in gene expression and the magnitude of GFP? Or magnitude of the HIV RNA measured in the RNAseq? The authors try to block some of the pathways observed in the transcriptomics data but do not observe the expected impact on GCB mediated induction of HIV expression in TFH. What do the authors propose as an explanation for these results?
2. For the in vitro experiments the authors use primary TFC, FDC and GCB derived from tonsils harvested from pediatric donors. Given the difference in immunological profile of Tfh cells between adolescents and adults, how generalizable are the observations made to adults in these experiments? Or are these observations specific to pediatric HIV infection? For example, it has been shown that Tfh cells exhibit age dependent altered tissue localization and transcriptional programming (*Immun Ageing*. 2014;11:12, *Nat Immunol*. 2023;24(7):1124–1137, *Elife*. 2021;10:e70554). Would the authors expect a similar result using adult derived tonsil MNCs? Although the authors use adult LN from men living with HIV on and off ART in the final figure to validate their findings, there is no discussion of how age related changes in GCB and TFH from their in vitro experiments would impact there conclusions to adult PWH.
