# Supplementary material for: Lymphoid B cells upregulate HIV-1 ex vivo and are linked to its expression in vivo
Source: PLoS Pathog. 2025 Dec 1;21(12):e1013661. doi: 10.1371/journal.ppat.1013661 (PMC12680345; doi:10.1371/journal.ppat.1013661)
Supplement: Attachment — Submitted filename: Response to Reviewers.docx [file ppat.1013661.s010.docx]

**Response to Reviewers**

Reviewer #1:

*This manuscript is really well written, designed and is investigating an interesting parameter often forgotten in the field which is the effect of B cells on infected cells.
I had great pleasure reading this manuscript, but I would like to raise a few concerns and make suggestions that hopefully can improve this interesting work.*

**We are grateful for the reviewer’s kind remarks and suggestions.**

1- Tonsils are a great tissue source however, if tonsillectomy is performed it is usually because of recurrent inflammation/infections. The authors are not raising this limitation in their discussion, and I think it would be important to mention that the levels of activation (in B and T cells) in those samples are likely not normal and can potentially affect results and increase cell death ex vivo and secretion of cytokines and other important factors.

**The reviewer’s points are well taken. We have amended the discussion to include this as a limitation beginning on line 387 of the marked-up copy of the manuscript.**

2- Sorry if I missed it in the method but, it was difficult for me to understand if ex vivo the isolated GCB cells and Tfh put together in culture were from the same donor or donors were mixed – as this can highly affect the response/activation of the cells.

**Cell cultures included only autologous cells. We have clarified this in the methods section and also added this information to the relevant figure legends.**

3- One important data missing in the ex vivo culture is the viability of the B cells- B cells aren’t the easiest cells to culture and are highly sensitive so can die rapidly which would explain the results obtained in culture and would also be dose (or ratio) dependent. The authors shown the viability of TFH but not of the GCB cells.

**As the reviewer noted, B cell viability in cell culture was relatively less than that of T cells, consistent with prior literature demonstrating that B cells are more fragile than T cells under these culture conditions.**

**These data have been added to the manuscript (Fig. S1B) and reported on line 106. GCB exhibit high levels of apoptosis and cell death *in vivo*, and consequently it is likely that TFH interact with dead or dying GCB *in vivo*. Thus, the increased GCB cell death in culture may at least partially replicate *in vivo* conditions. Importantly, GCB were viable upon placement in cultures and the cultures were of short duration, i.e., 3 days. Therefore, GCB had the opportunity to interact with TFH before they died. Furthermore, when GCB were physically separated from TFH (Fig. 1F), they did not significantly alter HIV expression, suggesting soluble factors relating to GCB cell death were not implicated in HIV expression. The strongest evidence against the notion that our findings were simply the result of GCB cell death is that GCB-enhanced HIV replication in culture was largely blocked by anti-MHC-II antibody (Fig. 2D).**

4- I was a little confused by the number of samples analyzed and the “n” reported in graphs – for example in the text the authors are talking about 6 samples being analyzed and flow data have often n= 7 or 1. Can the authors clarify this? Is it the flow analysis being repeated on same samples? If so can it be indicated on graphs if same sample is represented in different runs.

**All data points are independent biological replicates. We discovered only one instance of a discrepancy in Fig. S2I between the figure and figure legend. The text now reflects the correct n. Furthermore, in Fig. 2D, we emphasized that these data were from a separate set of experiments than the other graphs in Fig. 2.**

5- Regarding the in situ work, I have great concerns regarding the pattern of the representative pictures presented in figure S5 and Figure 6. First, the in situ CD4 is a single indicating a DNA signal (as all RNAprobe will detect complementary DNA but pattern will be a single dot) and not an expression of CD4 in that particular cell- I hope the authors took this into consideration while quantifying their cells of interest.

**While RNAscope can theoretically detect DNA, the probes are designed to hybridize with single-stranded RNA and the protocol is optimized to favor RNA detection. The assay includes protease digestion to expose RNA but does not include DNAse. Consequently, DNA, which is double-stranded and tightly packaged, is less accessible to the RNAscope probes, and requires longer incubation times and other modifications for reliable detection. In Fig. S5 in the manuscript, although a prominent CD4 RNA dot is shown, there are also less prominent dots present. If RNAscope detected CD4 DNA, then every cell in the figure should have two positive dots, but this was not observed. Prior to performing these studies we tested the accuracy of the CD4 RNA probe by analyzing several tissue specimens using both a CD4 antibody and the CD4 RNA probe. We found excellent concordance between the two assays. Thus, we do not believe that DNA staining was a significant contributor to the RNAscope detection of CD4 expression.**

The vRNA signal in figure S5 is quite unusual (figure 6 is more what is expected).

**The reviewer is correct that the vRNA signal is different in the two images. All images of RNAscope targets were taken using six 1µm z-stacks in this study. These targets included BCL6, CD4, and vRNA. For clear presentation in Fig 6, we used a single plane, and not the compressed z-stacked image of vRNA. For all other images, we showed the full z-stacked compressed image to clearly demonstrate expression of all targets. We have clarified when z-stacks were used and when a single plane was shown in the figure legends.**

Finally using in situ for BCL6 imply a need for membrane marker to determine an accurate segmentation of the cell and so being able to identify which dot belongs to which cells- I might have missed it, but I didn’t see anything regarding cell segmentation for accurate phenotyping in the method.

**To identify TFH for Figs. 5F and 5G, cells were stained with antibodies to CD3 and PD-1, and in situ hybridization was performed for BCL6 and CD4. Both CD3 and PD-1 are cell surface markers and only BCL6 and CD4 signals detected within those areas defined by CD3 and PD-1 were used to determine if a cell was a TFH. In Figure S5 we used a different approach. HIV RNA signal well defines the cytoplasm of cells and was used instead of cell surface marker staining to define localization of BCL6 within vRNA+ cells.**

6- The quantification of B cells in all patients, within and out of BCF needs to be added to this manuscript to see if in those patients the repartition of B cells and their pattern is similar before performing any comparison between group and tissue areas.

**We have added two new figures (Figs. 6E and F) demonstrating frequencies of B cells in follicular and extrafollicular regions, respectively. As described in the manuscript and consistent with the published literature, frequencies of B cells were significantly elevated in B cell follicles of PLWH. B cell frequencies within follicles tended to be higher in PLWH on ART compared to those not on ART, but differences were not statistically significant. In contrast, frequencies of B cells in extrafollicular regions were significantly lower in those on ART compared to those not on ART (Fig. 6F). In Fig. 6I, the data are adjusted by CD3+CD4+ cell frequencies measured in the tissue sample (as shown in Fig. S5). In Fig. 6J, the probability of an adjacent B cell is calculated (as described in methods) using the extrafollicular B cell frequencies shown in Fig. 6F. Thus, the data were already adjusted in both figures according to cell frequencies.**

7- There is a great difference in cell density in BCF (especially GC) and T cell zone or medullary cords and this can change drastically with inflammation and treatment. Did the authors performed nuclei count and can express their data per million nuclei instead of mm2 of tissue to see if the conclusion and patterns stay similar.

**Frequencies of total cells were determined after counting all cell nuclei (DAPI+ cells) within 10 areas of approximately 50 cells randomly selected in up to three nonadjacent sections for the extrafollicular compartment and 4 areas of approximately 80 cells in the follicular compartment. Results are shown below. No statistically significant differences were observed in cell density within either B cell follicles or extrafollicular regions relating to use of ART in PLWH. In PLWH, cells in follicles were more dense than in extrafollicular regions, but the difference was relatively small, approximately 1.2 fold. No such difference was observed in PLWH not on ART. We do not believe that our findings should be adjusted by these data given the minimal differences observed. We have not added these data to the manuscript, but are willing to include them as an additional supplemental figure if the editor wishes.**

8- Running a duplex (vRNA+vDNA) would have been more accurate and powerful to compare treated and not treated patients with the hypothesis of B cells increasing genome expression.

**We considered the studies suggested by the reviewer, but ultimately decided not to pursue that avenue for the following reasons: 1) much of the integrated DNA found in PLWH is defective, and in treated individuals the proportion of defective DNA increases with time. We thought it was unlikely that we would be able to detect the anticipated differences given the large background of defective HIV DNA. 2) To our knowledge, *in situ* assays to detect intact vRNA are not generally available, although they are currently under development. Once those assays are available, we agree with the reviewer that this would be an important avenue to pursue. We have added this point to the discussion as a future direction beginning on line 403 of the marked-up version.**

**Reviewer #2:**

1. In Figure 2 the authors perform transcriptomics analysis on TFH cells in vitro infected with X4 or R5 tropic HIV in the presence or absence of GCB. Although the authors show the data in Figure 2 and report the gene expression measurements in xcel files, the authors seem to be leaving it up to the reader to review and interpret their findings. There is really no discussion of the findings in the manuscript other than to note a few genes in lines 128-131. The authors should include a more detailed discussion on the gene signatures they are showing in Figure 2 and more discussion on the significance of these findings in their conclusions.

**We have added more discussion on how these genes/pathways fit in the context of the observed MHC-II blockade results beginning on line 307 of the marked-up copy of the manuscript.**

Do the authors observe any correlations between the magnitude of the change in gene expression and the magnitude of GFP?

**We believe that the study is underpowered to make correlations between magnitude of changes in gene expression and GFP, and that there is risk of arriving at both false positive and false negative results.**

Or magnitude of the HIV RNA measured in the RNAseq?

**We did not perform RNAseq assays.**

The authors try to block some of the pathways observed in the transcriptomics data but do not observe the expected impact on GCB mediated induction of HIV expression in TFH. What do the authors propose as an explanation for these results?

**One possible explanation is that the induction of HIV expression occurs at an earlier stage prior to activation of these gene pathways. We have added this explanation to the discussion beginning at line 307 of the marked-up copy of the manuscript.**

2. For the in vitro experiments the authors use primary TFC, FDC and GCB derived from tonsils harvested from pediatric donors. Given the difference in immunological profile of Tfh cells between adolescents and adults, how generalizable are the observations made to adults in these experiments? Or are these observations specific to pediatric HIV infection? For example, it has been shown that Tfh cells exhibit age dependent altered tissue localization and transcriptional programming (Immun Ageing. 2014;11:12, Nat Immunol. 2023;24(7):1124–1137, Elife. 2021;10:e70554). Would the authors expect a similar result using adult derived tonsil MNCs? Although the authors use adult LN from men living with HIV on and off ART in the final figure to validate their findings, there is no discussion of how age related changes in GCB and TFH from their in vitro experiments would impact there conclusions to adult PWH and this should be noted and discussed.

**The reviewer raises an important point. Tonsil cells from children are a readily accessible and convenient source of Tfh, but there are limitations to this model. Well established age differences in Tfh are a definite limitation. Because we do not have tonsils from adults to use as a comparator, we can only speculate as to whether findings in adults would be similar. This is an important limitation and we have added it to the discussion beginning on line 389 of the marked-up copy of the manuscript. Nevertheless, we do believe that findings in tissue analyses regarding the adjacency of B cells to vRNA+ cells provide circumstantial evidence that the mechanisms seen *ex vivo* occur *in vivo*.**

**Many thanks to the reviewers for their thoughtful suggestions, which we believe have improved the manuscript. We hope that the revised manuscript is acceptable to the editor.**

**Sincerely,**

**Matthew Ollerton, PhD on behalf of the manuscript authors**
